# Associations of age, BMI, and renal function to cortisol after dexamethasone suppression in patients with adrenal incidentalomas

**DOI:** 10.3389/fendo.2022.1055298

**Published:** 2023-01-16

**Authors:** Henrik Olsen, Martin Olsen

**Affiliations:** ^1^ Department of Clinical Sciences in Lund, Lund University, Lund, Sweden; ^2^ Department of Internal Medicine, Ängelholm Hospital, Ängelholm, Sweden; ^3^ Department of Business Development and Technology, Aarhus University, Aarhus, Denmark

**Keywords:** adrenal incidentaloma (AI), autonomous cortisol secretion (ACS), GFR, BMI - body mass index, age, artificial intelligence, explainable AI

## Abstract

**Introduction:**

The specificity of cortisol after 1 mg dexamethasone (cortisol_DST_) ≥50 nmol/L as a criterion for mild autonomous cortisol secretion (MACS) is approximately 85% in patients with adrenal incidentalomas (AI). The aim was to study the associations of cortisol_DST_ to age, BMI, and renal function.

**Methods:**

We studied 1,129 patients with AI examined from 2005 to 2015 at Skåne University Hospital and Helsingborg Hospital. The covariates studied were gender, age, BMI, estimated glomerular filtration rate (eGFR), treatment with inhalation steroids, size of the AI, and size of the smallest AI in patients with bilateral AI (set to 0 in unilateral AI). We used machine learning models to uncover potential nonlinear associations. They were trained to fit the data and examined using feature importance analysis and partial dependence plots. Partial dependence plots show the marginal effect on cortisol_DST_ of a covariate averaging over other covariates.

**Results:**

Cortisol_DST_ was strongly associated with the size of the AI and weakly associated with age, BMI, and eGFR according to the feature importance analysis. The partial dependence plots indicated relatively linear relationships for cortisol_DST_ to age (positively) and eGFR (negatively). The association between cortisol_DST_ and BMI was nonlinear. At BMI below 30 kg/m^2^, cortisol_DST_ was negatively associated with BMI, but it was unchanged at higher BMI levels. Using linear regression, we found that cortisol_DST_ increased by 11% (95% CI, 7%–14%) for each 10-year increase in age. In patients with a BMI below 30 kg/m^2^, cortisol_DST_ increased by 23% (95% CI, 16%–31%) for each 5 kg/m^2^ decrease in BMI. We found no association at BMI levels above 30 kg/m^2^. Cortisol_DST_ increased by 9% (95% CI, 6%–11%) for each 10 ml/min/1.73m^2^ decrease in eGFR.

**Conclusions:**

Cortisol_DST_ is positively associated with age, negatively with BMI if below 30 kg/m^2^, and negatively with eGFR. These associations should be considered before diagnosing MACS.

## Introduction

An adrenal incidentaloma (AI) is defined as an adrenal enlargement detected at an imaging examination performed for another indication than suspected adrenal disease. The prevalence of adrenal enlargements is 3.5% in the population at ages above 60 years, and AIs are thus frequently found at cross-sectional imaging of the upper abdomen (1). Patients with AIs may have mild autonomous cortisol secretion (MACS), which should be suspected when cortisol after 1-mg dexamethasone suppression (cortisol_DST_) is ≥50 nmol/L (2). MACS is associated with metabolic complications, cardiovascular disease, and increased mortality, so an accurate diagnosis is important (3).

Cortisol_DST_ is ≥50 nmol/L in approximately 50% of patients with AI, but the specificity for MACS is only approximately 85% (3–6). The false-positive results may have different causes. An established explanation is inadequate suppression of the HPA axis due to low dexamethasone concentrations (7). Other putative causes are differences in cortisol_DST_ levels related to gender, age, BMI, and GFR (5, 6, 8–11). These reported associations of cortisol_DST_ to gender, age, BMI, and GFR have, however, not been studied simultaneously in a large cohort of patients. Furthermore, the associations have often been studied with linear or logistic regression, which does not detect nonlinear associations. Recently, the field of explainable artificial intelligence has gained popularity, including partial dependence and feature importance analysis. Analysis of the partial dependence of the covariates for a variety of machine learning (ML) models provides the possibility to detect nonlinear relationships, and feature importance analysis can be used to estimate how important a covariate is for a prediction. Understanding the associations between factors not linked to the incidentalomas and cortisol_DST_ may result in a more precise evaluation of whether the AI is cortisol-secreting or not. This could possibly lead to a lower number of falsely elevated cortisol_DST_. We, therefore, studied these associations with ML and conventional statistical methods in a large cohort of patients with AI.

The aim was to study the associations of cortisol_DST_ after dexamethasone with gender, age, BMI, and renal function in patients with adrenal incidentalomas.

## Methods

### Patients

Patients investigated for AI at the Department of Endocrinology, Skånes University Hospital, and Department of Internal Medicine, Helsingborg Hospital, Sweden, between 1 January 2005 and 15 September 2015, were screened. Data were not collected after 15 September 2015, due to an exchange of our cortisol assay. Exclusion criteria were metastatic malignancy, AI <1 cm, AIs considered not to be adenomas, such as cysts, myelolipomas, haemorrhage, pheochromocytomas, primary hyperaldosteronism, or clinical Cushing syndrome, oral corticoid treatment with more than single doses in the past 3 months, treatment with systemic oestrogen, and medication affecting dexamethasone metabolism. Medical history was collected from patient records. Patients were given 1 mg of dexamethasone orally at 11:00 PM, and cortisol levels were determined at 8:00 AM the following morning (cortisol_DST_). Plasma Adrenocorticotropic hormone (ACTH) was measured in a subset of patients at 8:00 AM, but in Malmö until 22 February 2012, it was analysed with a relatively inaccurate method (Nichols). Patients were screened for primary aldosteronism and pheochromocytoma. The study was approved by the Ethics Committee, Lund, Sweden.

### Imaging

The size of the AIs was defined as the maximal axial diameter on a CT scan.

### Analyses

Plasma cortisol was analysed using an immunoassay (Cobas, Roche Diagnostics, Mannheim, Germany). The reference range was 171 to 536 nmol/L, and the coefficient of variation was 2.1% at 94.9 nmol/L. Plasma creatinine was analysed with an IDMS-standardized enzymatic colourimetric assay (Cobas, Roche Diagnostics). Plasma ACTH was analysed using a two-step immunometric sandwich assay (Cobas, Roche Diagnostics), where the reference range was 1.6 to 13.9 pmol/L, the coefficient of variation was 5.4% at 1.1 pmol/L, and the detection limit was 0.23 pmol/L.

### Calculation of estimated glomerular filtration rate

The estimated glomerular filtration rate (eGFR) was calculated using the MDRD expression but without using a correction for ethnicity (12). Levels of eGFR calculated to be higher than 90 ml/min/1.73m^2^ was set to 90 ml/min/1.73m^2^ in the calculations and in the plots.

### Covariates

We studied the associations to gender, age, BMI, eGFR, treatment with inhalation steroids, the size of the AI, and, if bilateral, the largest and size of the second AI. In patients with unilateral AI, the size of the second AI was set to 0 mm. Smoking was not included in the model since the association between smoking and cortisol_DST_ may be caused by selection bias and not being causal (13). However, smoking was included in a linear regression analysis of the whole patient cohort to show whether this would significantly alter the estimates of the other covariates.

### Statistical methods

#### Machine learning models

A variety of seven different types of ML models were trained using supervised learning to produce nonlinear models for predicting cortisol_DST_. The natural logarithm of the cortisol level (ln-transformed cortisol_DST_) was used in all calculations since the associations to the covariates were much stronger than without this transformation. The nonlinear models used were AdaBoost, gradient boosting, extreme gradient boosting, k-nearest neighbours, multilayer perceptrons, support vector regression, and random forests. A standard linear regressor was also constructed for comparison. To uncover patterns between the covariates and the logarithm of the cortisol level, the models were examined using two techniques within the field of explainable AI: feature importance analysis and partial dependence plots—these techniques will now be explained in broad terms (14).

The purpose of feature importance analysis is to figure out which of the covariates is the most important for determining the cortisol level. We used permutation feature importance since this type of feature importance can be used for any ML model. To compute the permutation importance of a model for a specific covariate, you simply measure the drop in performance for the model if you permute the values of the covariate for all the patients.

A univariate partial dependence plot (PDP) for a particular model is used to illustrate the marginal dependence on the logarithm of the cortisol level for a single covariate. Let us look, as an example, at the way a PDP for the BMI covariate is constructed. In the first step, we change the BMI for all patients to a certain value—for example, 30. In the second step, we compute the average of the logarithm of the cortisol level for all the patients with their BMI fixed at 30. We then repeat this process for other values than 30 and construct the PDP. In other words, the PDP shows the marginal effect of the BMI averaging over all the other features in an “all else equal” fashion (ceteris paribus in Latin). Please note that we are not computing the average (ln) cortisol level for the model for a patient with a BMI of 30. Bivariate PDPs are produced by fixing the values of two covariates in the first step described previously.

The models and techniques were implemented in Python using the popular library scikit-learn except for extreme gradient boosting where we used the XGBoost library. The dataset was split into a training set and a test set using an 80/20 split. For each ML model, we created a grid of potential configurations for the model. We then performed a random search on the grid, considering up to 200 configurations, and picked the configuration with the highest *r* ^2^ score using cross-validation on the training set (the scikit-learn function RandomizedSearchCV was applied). The permutation importance was computed using the test set. Standard box plots were produced for the permutation importance, with boxes representing the interquartile range (IQR) and a line representing the median for 20 random permutations per covariate. Default settings (1.5 × IQR) were used for the whiskers in the box plots.

### Conventional statistics

Results are given as a median and interquartile range. Multivariate linear regression was used to study the associations of ln-transformed cortisol_DST_ to the covariates adjusted for the remaining covariates. Multivariate logistic regression was used to study the associations of cortisol_DST_ to be ≥50 nmol/L to the covariates adjusted for the remaining covariates. Segmented multivariate linear regression was used to study the associations of ln-transformed cortisol_DST_ to age, BMI, eGFR, size of the AI, and size of the second AI at different ranges of the variates to present further proof of nonlinear associations suggested by the ML models. Restricted cubic splines were used to plot the predicted probabilities for cortisol_DST_ to be ≥50 nmol/L in relation to the continuous covariates adjusted for the remaining covariates. The intraclass correlation coefficient (ICC) was calculated using mixed models (15). The covariance structure for repeated measures was identity, and for random effects, it was unstructured. Outliers were not excluded.

## Results

We screened 1,593 patients and excluded 464 according to the exclusion criteria. We thus studied 1,129 patients, of whom 180 had bilateral AIs. The prevalence of cortisol_DST_ ≥50 nmol/L was 46% in the cohort. The characteristics of the patients divided into two groups according to whether cortisol_DST_ was <50 nmol/L or cortisol_DST_ was ≥50 nmol/L are given in **Table 1**.

**Table 1:** Patient characteristics.

Patient characteristics	Cortisol_DST_ <50 nmol/L (*n* = 613)	Cortisol_DST_ ≥50 nmol/L (*n* = 516)
Age (years)	63.2 (55.1–69.7)	67.3 (61.0–74.4)
Female (*n* (%))	352 (57)	322 (62)
BMI (kg/m^2^)	28.2 (25.3–32.3)	26.8 (23.3–30.5)
eGFR (ml/min/1.73 m^2^)	88 (77–≥90)	80 (66–≥90)
Current smoker (*n* (%))	187 (31)	226 (44)
Comorbidities		
Cardiovascular disease (*n* (%))	110 (18)	131 (25)
Treatment with inhalation of steroids	55 (9)	49 (9)
Hormones		
Cortisol_DST_ (nmol/L)	34 (27–41)	75 (59–102)
ACTH pmol/L^a^ (*n* (%))	3.7 (2.5–5.4)	2.1 (1.4–3.9)
Imagining		
AI size^b^ (mm)	18 (14–24)	24 (17–30)
Bilateral AI (*n* (%))	64 (10)	116 (22)
AI size second AI in bilateral AI (mm)	14 (12–18)	20 (15–25)

a ACTH analyses with the Roche method were available in 601 patients.

b If bilateral AI, the size of the largest AI is given.

### Associations to cortisol_DST_

Ln-transformed cortisol_DST_ was used in the models since the associations to the covariates were much stronger than without this transformation, as previously stated in the Methods section. **Table 2** presents the *r* ^2^ scores for the ML models and linear regression for the training set without cross-validation, the training set with cross-validation, and the test set. The ML models are not performing better than the linear regression model predicting the cortisol_DST_ level. As we will see later, the ML models play a crucial role by uncovering nonlinear relationships and by supporting that there is a linear relationship between the ln-transformed cortisol_DST_ and some of the covariates.

**Table 2:** The *r* ^2^ scores for seven ML models and linear regression.

*r* ^2^ score	Adaboost	GB	XGB	k-NN	MLP	SVR	RF	Linear regression
Training set	0.31	0.36	0.41	1.0	0.36	0.29	0.44	0.30
Training set cross-validation	0.18	0.24	0.24	0.22	0.25	0.24	0.24	–
Test set	0.21	0.27	0.27	0.24	0.27	0.28	0.27	0.28

**Figure 1:**
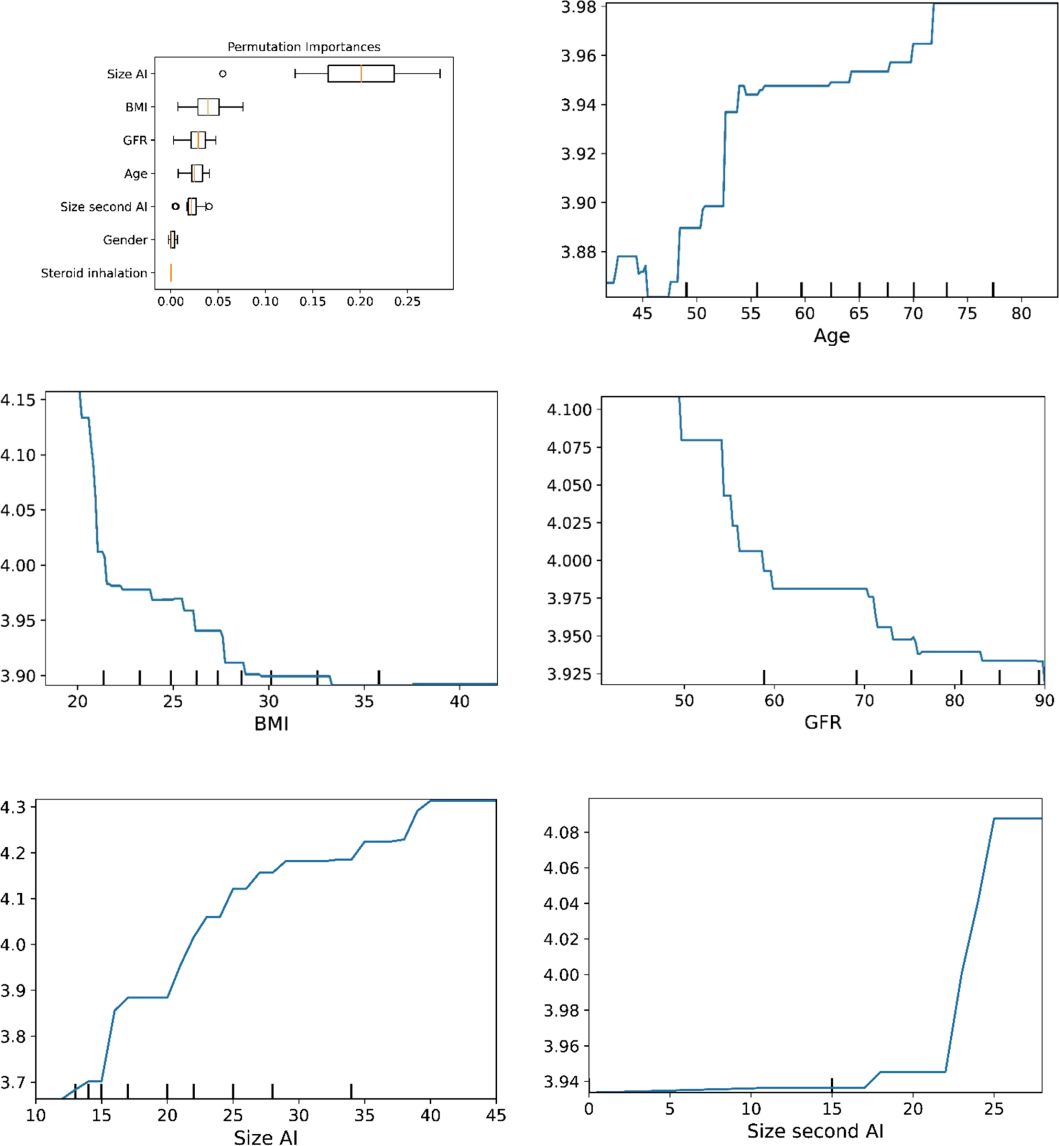
The plots for permutation importance and ln-transformed cortisol_DST_ against age, BMI, eGFR, size of AI (in bilateral AI, the size of the largest AI), and size of the second AI using the ML model AdaBoost.

**Figure 2:**
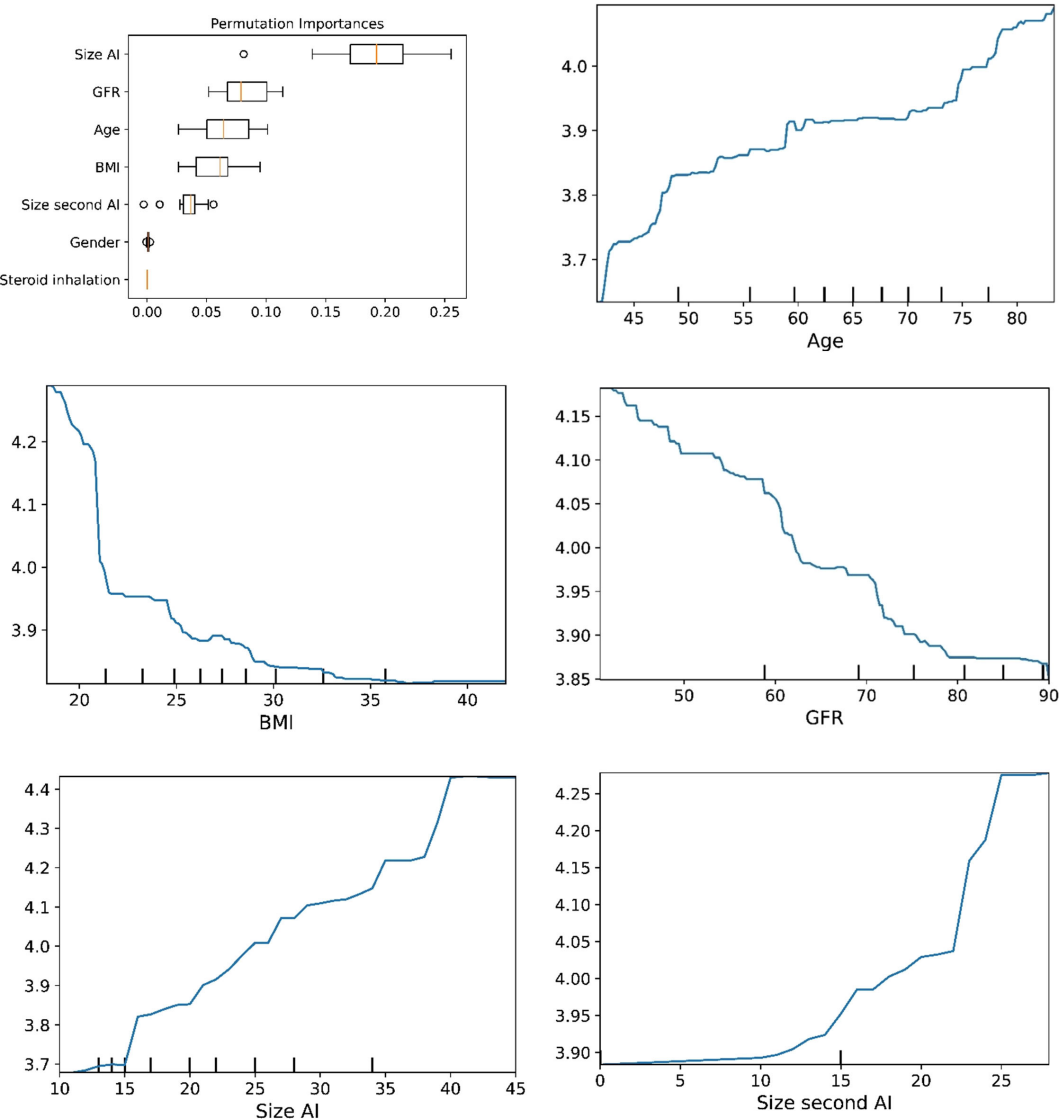
The plots for permutation importance and ln-transformed cortisol_DST_ against age, BMI, eGFR, size of AI (in bilateral AI, the size of the largest AI), and size of the second AI using the ML model gradient boosting.

**Figure 3:**
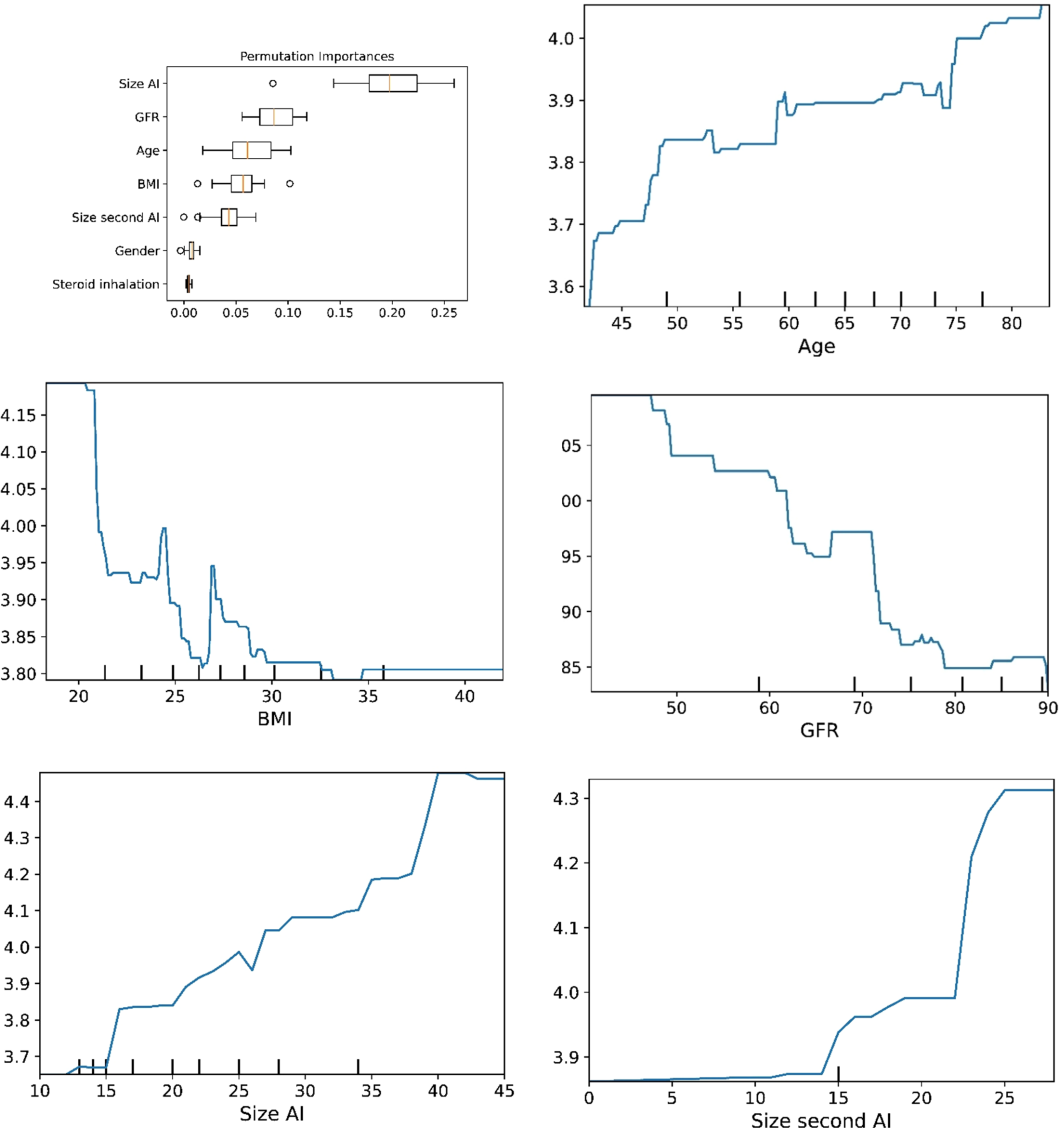
he plots for permutation importance and ln-transformed cortisol_DST_ against age, BMI, eGFR, size of AI (in bilateral AI, the size of the largest AI), and size of the second AI using the ML model extreme gradient boosting.

**Figure 4:**
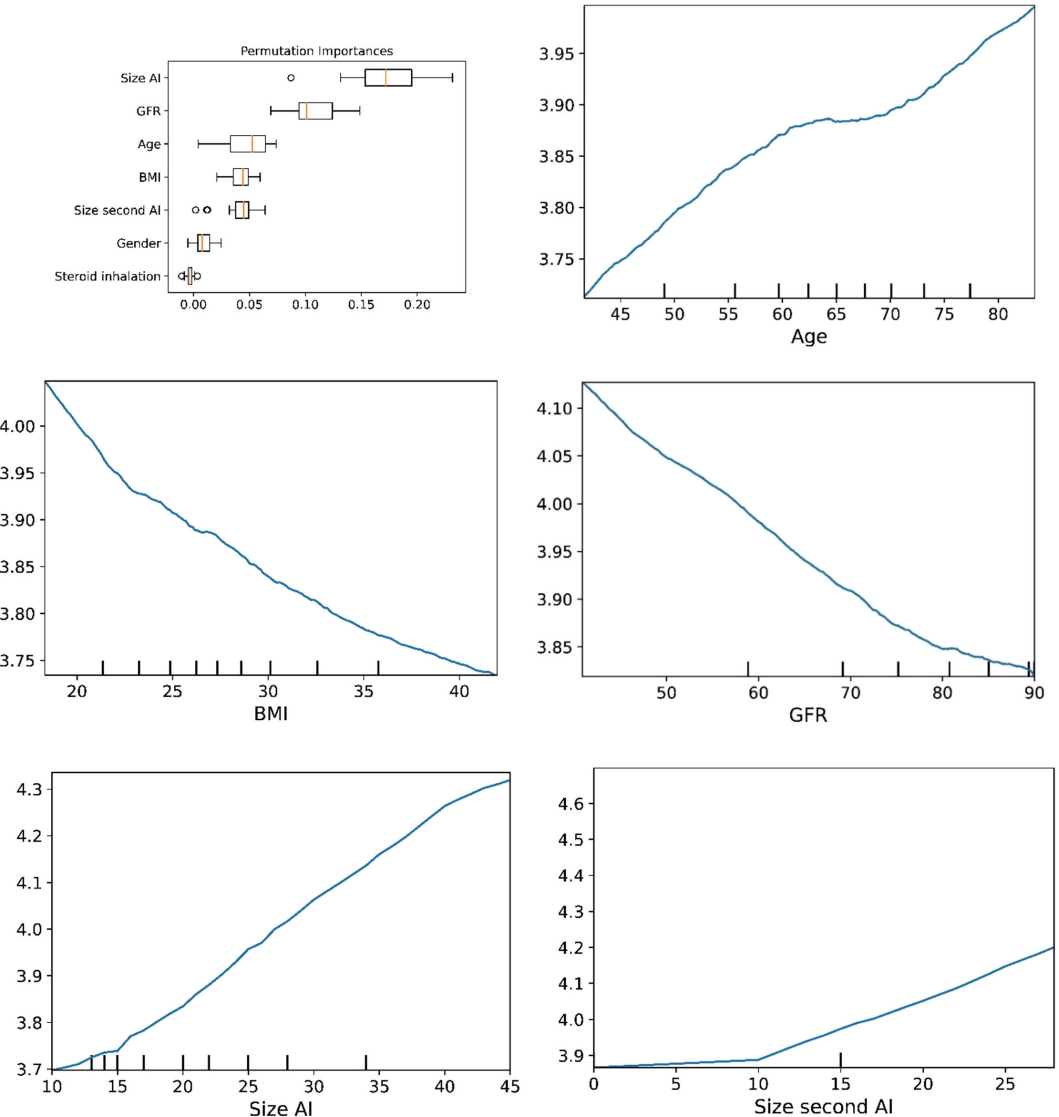
The plots for permutation importance and ln-transformed cortisol_DST_ against age, BMI, eGFR, size of AI (in bilateral AI, the size of the largest AI), and size of the second AI using the ML model K-nearest neighbours.

**Figure 5:**
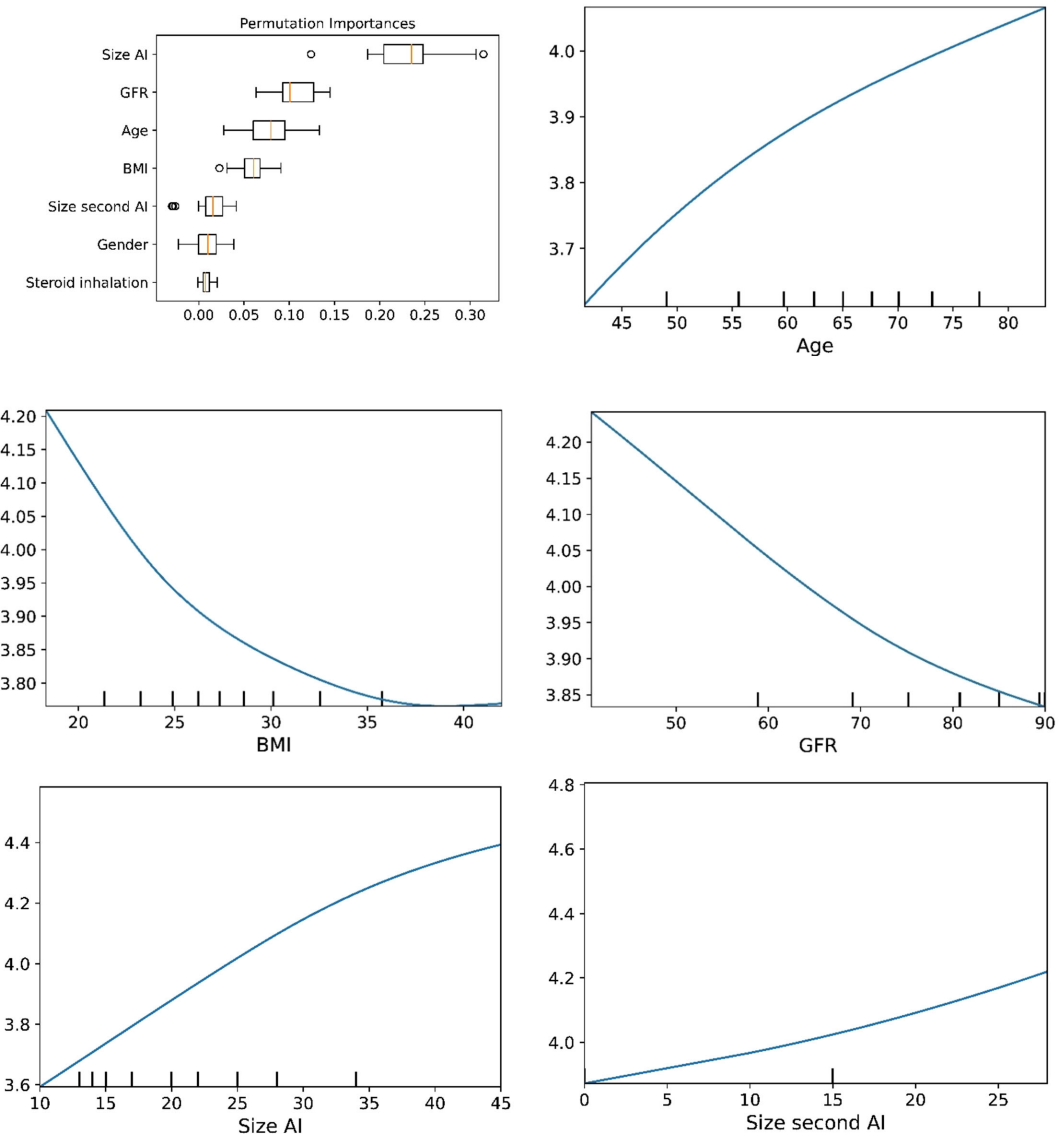
The plots for permutation importance and ln-transformed cortisol_DST_ against age, BMI, eGFR, size of AI (in bilateral AI, the size of the largest AI), and size of the second AI using the ML model multilayer perceptron.

**Figure 6:**
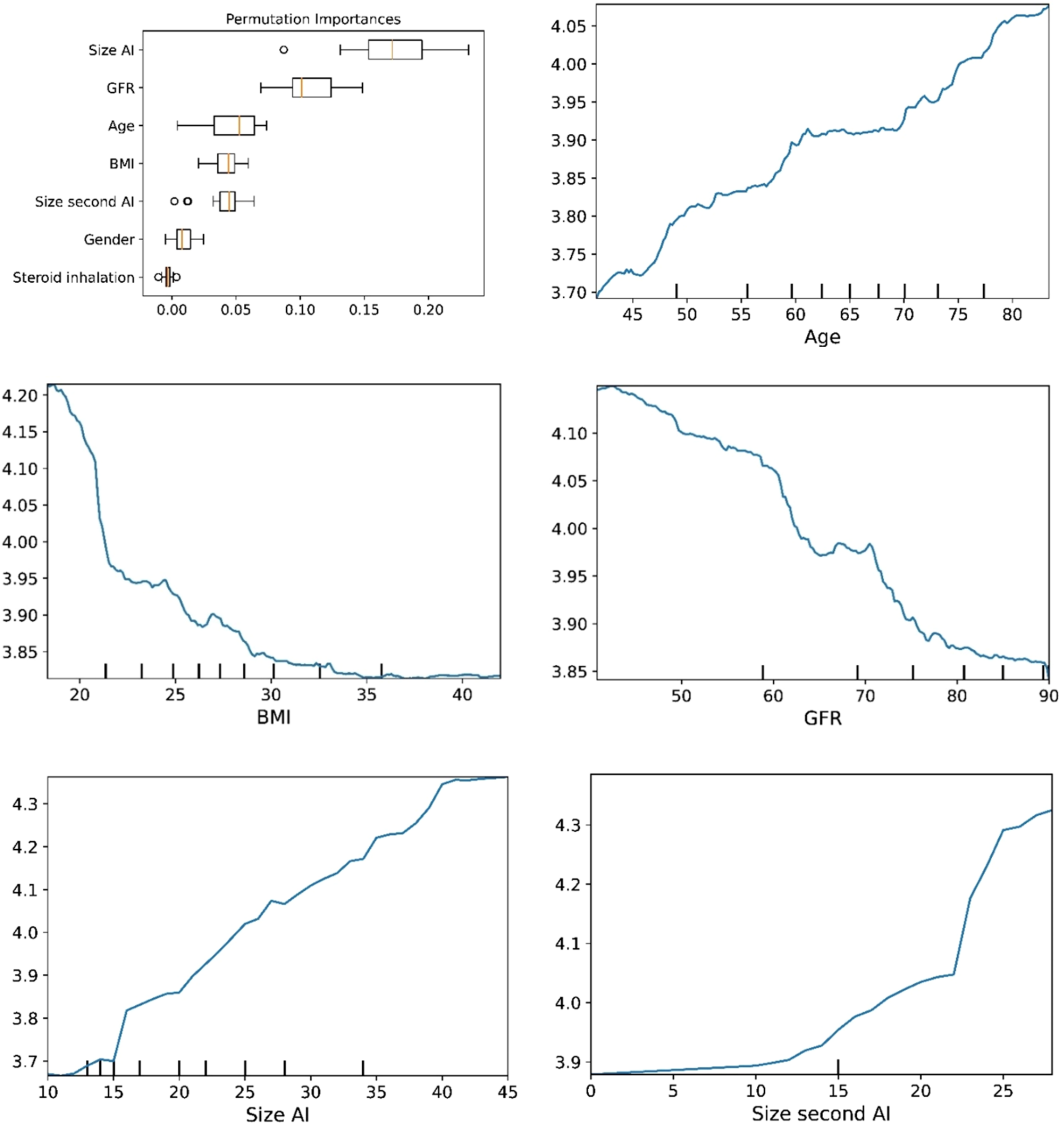
The plots for permutation importance and ln-transformed cortisol_DST_ against age, BMI, eGFR, size of AI (in bilateral AI, the size of the largest AI), and size of the second AI using the ML model random forest. **Figures 1**–**6** The figures give the found association to the studied variates at their levels between the 2nd and 98th percentiles. Random forests and gradient boosting presented a reasonable “smoothening” of the curve, and the slope of the curves resembled the slope of the curves from most of the other models. The slope for the relations between ln-transformed cortisol_DST_ to age, BMI, and eGFR were relatively similar in the models. However, AdaBoost found a weaker association between ln-transformed cortisol_DST_ and age and eGFR compared to the other six models.

**Figure 7:**
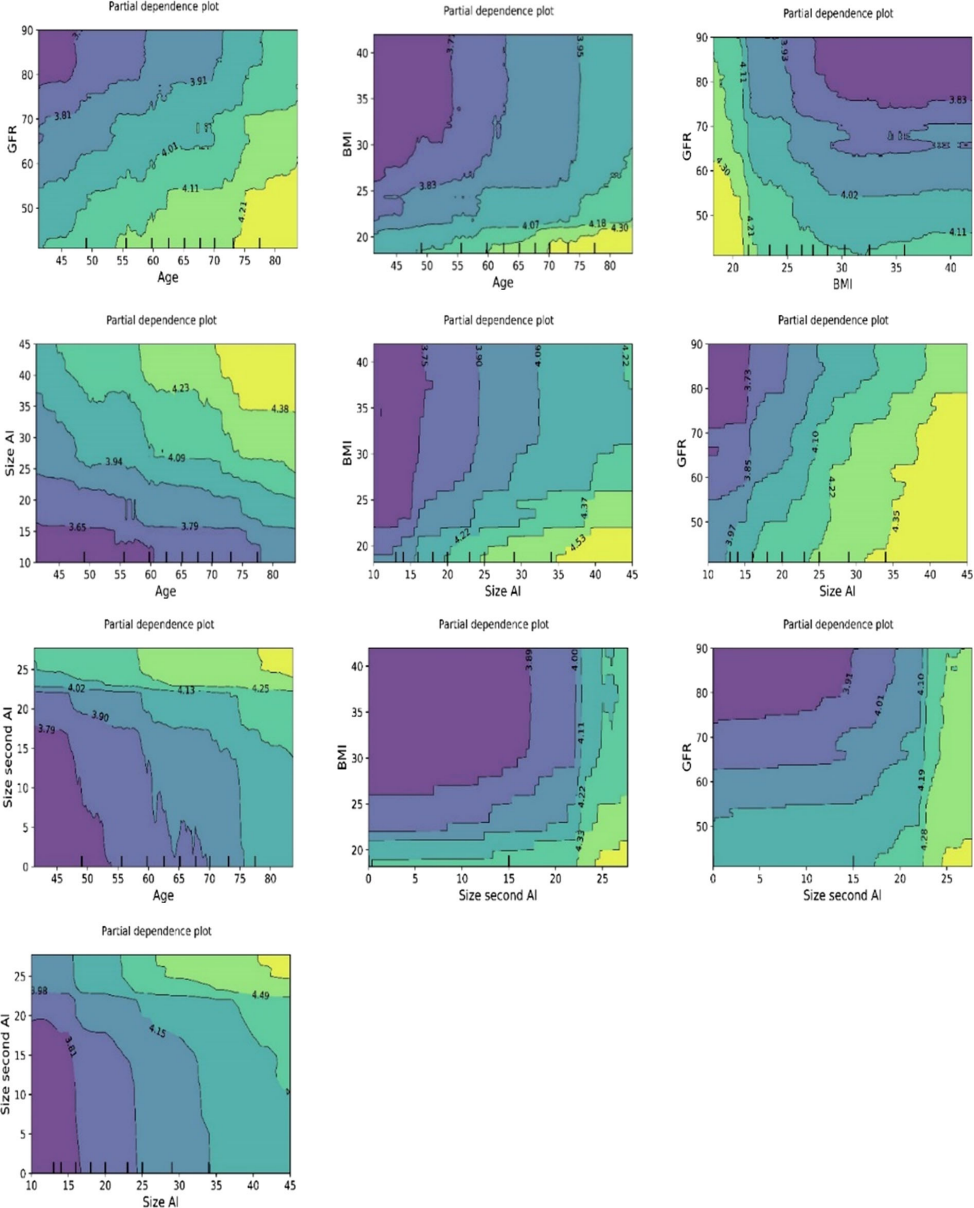
Bivariate plots of ln-transformed cortisol_DST_ developed using the ML model random forest. The three plots on the upper “row” of plots show the associations of ln-transformed cortisol_DST_ to two of the variates age, BMI, and eGFR. The associations of two of these to ln-transformed cortisol_DST_ seem to be addictive.

The plots for permutation importance and partial dependency for ln-transformed cortisol_DST_ against age, BMI, eGFR, the size of AI, and the size of the second AI for the different ML models are presented in **Figures 1**–**6**. A linear kernel was chosen for the support vector regression model during the randomized search process, resulting in a linear partial dependency, so the plots for this model are not included. The calculated permutation importance shows that the associations for age, BMI, eGFR, size of AI, and size of second AI are significant. The size of the AI has the strongest association with cortisol_DST_, and eGFR has the second-strongest association. The associations between age, BMI, and size in the second AI are weaker. The partial dependency plots show the association of each of the numerical covariates to cortisol_DST_. Age was positively associated with cortisol_DST_, and the association was relatively linear. eGFR was negatively associated with cortisol_DST_, and this association was also relatively linear. BMI was negatively associated with cortisol_DST_ at levels below 30 kg/m^2^. On the other hand, we noted no relation at BMI levels above 30 kg/m^2^. The association between size and cortisol_DST_ was positive and relatively linear. Cortisol_DST_ was approximately similar in patients with unilateral AIs (the second AI was 0 mm) and in patients with a second AI of less than 15 mm, but increased with size if larger. The bivariate partial dependency plots are presented in **Figure 7**. There were no clear signs that the associations between the variates and cortisol_DST_ were dependent on each other. Gender and treatment with inhalation steroids were not associated with cortisol_DST_.

### Linear regression, logistic regression, and restricted cubic splines

We studied the associations with conventional statistics to support the associations found using ML. The associations between cortisol_DST_ and Cortisol_DST_ ≥50 nmol/L are given in **Table 3**. Cortisol_DST_ and Cortisol_DST_ ≥50 nmol/L were both positively associated with female gender, age, size of the AI, and size of the second AI and negatively to BMI and eGFR. Cortisol_DST_ seems not to be different in patients treated with inhalations of steroids. *R* ^2^ for the relation of cortisol_DST_ to the covariates is 0.295. When the size of the AI and the size of the second AI were omitted, *R* ^2^ was only 0.133. The regression coefficients found at linear regression for age and eGFR correspond well with the slope of the association found with the ML methods.

**Table 3:** Associations to cortisol_DST_ and cortisol_DST_ to be ≥50 nmol/L in the whole cohort.

	Change in cortisol_DST_ (%)	Change in cortisol_DST_ (%)	OR for cortisol_DST_ ≥50 nmol/L	OR for cortisol_DST_ ≥50 nmol/L
Female vs. male	7 (1–14)	6 (−1–12)	1.50 (1.14–1.96)	1.42 (1.08–1.87)
Age (increase 10 years)	11 (7–14)	12 (9–16)	1.38 (1.20–1.59)	1.47 (1.28–1.70)
BMI (increase 5 kg/m^2^)	10 (7–12)	8 (5–11)	1.39 (1.23–1.57)	1.34 (1.18–1.51)
eGFR (decrease 10 ml/min/1.73 m^2^)	9 (6–11)	9 (7–12)	1.46 (1.31–1.62)	1.50 (1.35–1.68)
Treatment with inhalation steroids	−9 (−19 to −0.004)	−11 (−20 to −1)	0.86 (0.54–1.35)	0.78 (0.49–1.24)
Adenoma size (increase 10 mm)	27 (23–32)	26 (21–30)	2.00 (1.70–2.37)	1.94 (1.64–2.30)
Adenoma size of the second AI (increase 10 mm)	12 (8–17)	11 (7–16)	1.74 (1.40–2.16)	1.66 (1.34–2.06)
Smoking (yes vs. no)	–	19 (11–27)	–	1.94 (1.44–2.61)

The associations of the covariates to cortisol_DST_ and to cortisol_DST_ being ≥50 nmol/L are shown in two different calculations. In the first, smoking is not included as a covariate and in the second smoking is included. The associations of cortisol_DST_ and cortisol_DST_ being ≥50 nmol/L to the other covariates are similar when smoking is included and when smoking is not included in the calculations.

### Segmented regression

**Table 4** presents results for segmented regression of the covariates. Based on the assumption of a difference in the association between cortisol_DST_ and BMI at BMI levels below and above 30 kg/m^2^, according to the partial dependence plots, we performed segmented regression in these two groups. BMI levels <30 kg/m^2^ were negatively associated with cortisol_DST_, whereas it was not associated with levels ≥30 kg/m. We also performed segmented regression on the other covariates, and the results gave no clear indication of nonlinearity in any of these.

**Table 4:** Results from segmented regression.

	Change in cortisol_DST_ (%)
Age at age <64.3 years (increase 10 years)	15 (7–23)
Age at age ≥64.3 years (increase 10 years)	14 (6–23)
BMI at levels <30 kg/m2 (decrease 5 kg/m2)	23 (16–31)
BMI at levels ≥30 kg/m2 (decrease 5 kg/m2)	2 (−4–8)
eGFR at levels <79 ml/min/1.73 m2 (decrease 10 ml/min/1.73 m2)	10 (6–15)
eGFR at levels 79-90 ml/min/1.73 m2 (decrease 10 ml/min/1.73 m2)	5 (−7–18)
Adenoma size at sizes 10-20 mm (increase 10 mm)	39 (22–58)
Adenoma size at sizes ≥21 mm (increase 10 mm)	22 (14–30)
Bilateral AI with minor AI sized 10-15 mm vs. unilateral AI	7 (−5–20)
Bilateral AI with minor AI sized ≥16 mm vs. unilateral AI	34 (19–50)

The results are given only for the segmented variate. The given regression coefficients are adjusted for the remaining covariates.

### Associations to cortisol_DST_ ≥50 nmol/L

**Figure 8:**
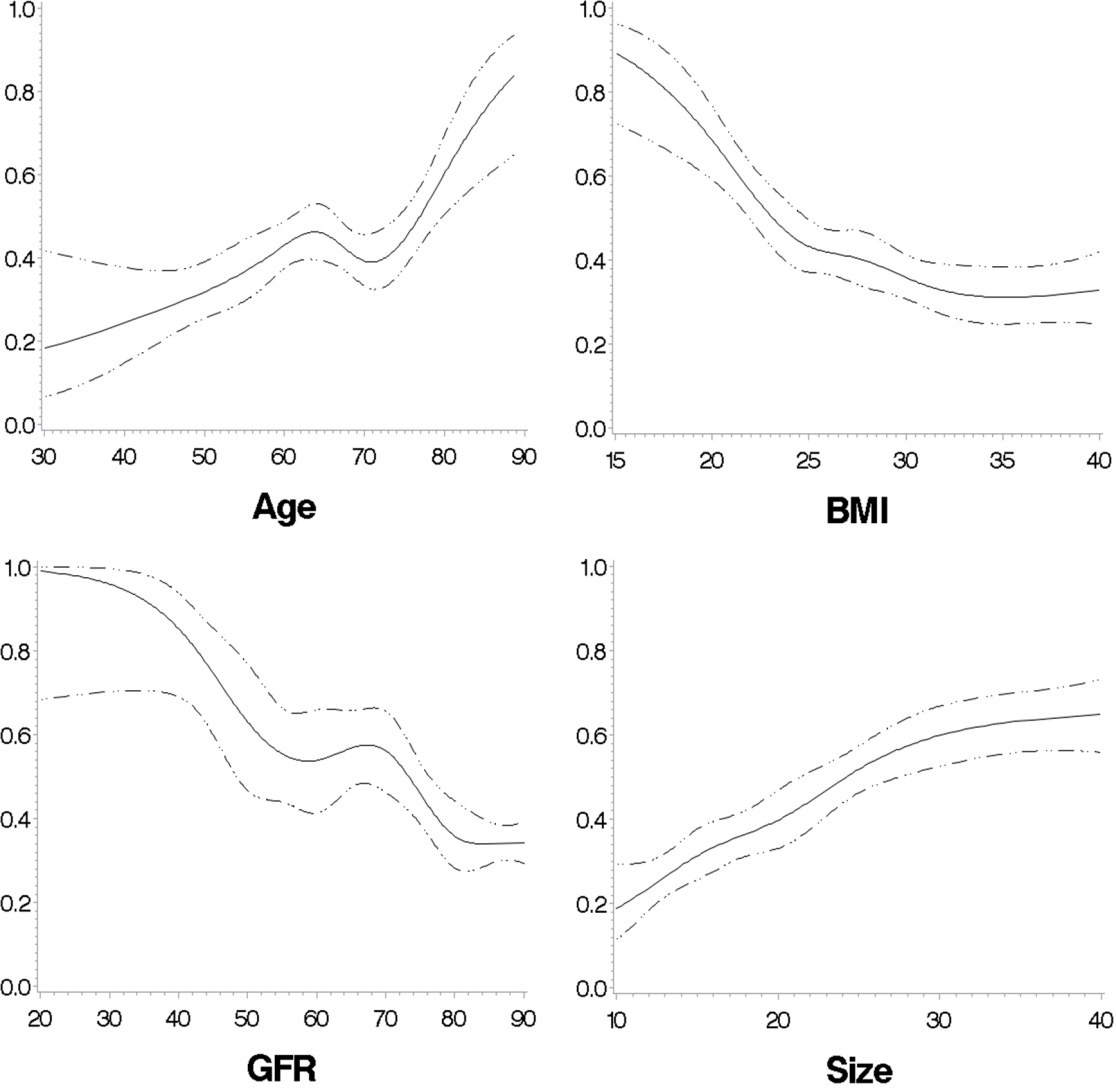
The predicted probabilities for cortisol_DST_ to be ≥50 nmol/L in relation to age, BMI, eGFR, and size of the AI in patients with unilateral AI. The figures are calculated with restricted cubic splines, and each of the variates is adjusted for the remaining three of the four variates.

The predicted probabilities for cortisol_DST_ ≥50 nmol/L in relation to age, BMI, eGFR, and size of the AI are shown in **Figure 8**.

### The probability for cortisol_DST_ ≥50 nmol/L in relation to the size of unilateral AIs in two patient groups with low and high probability depending on age, BMI, and eGFR

**Figure 9:**
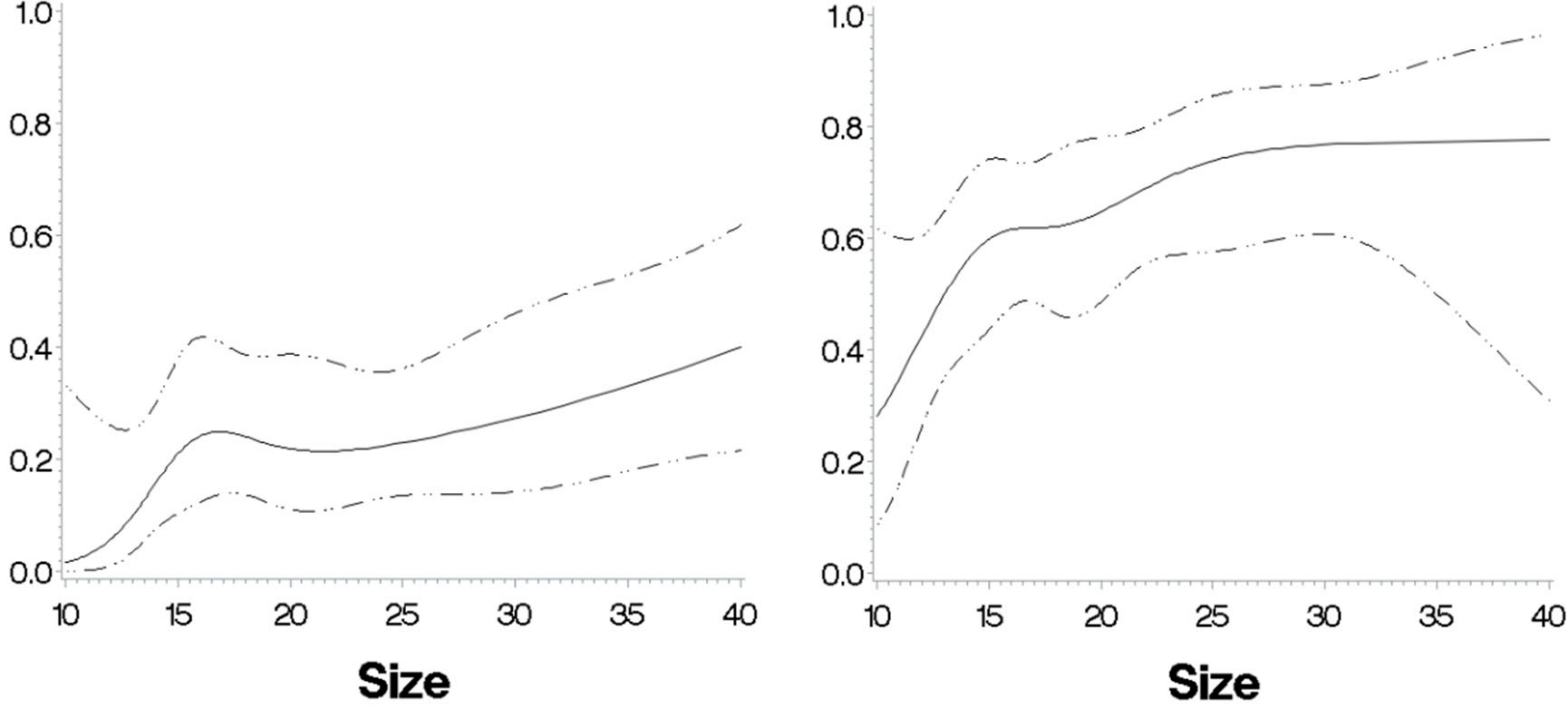
The predicted probabilities for cortisol_DST_ to be ≥50 nmol/L in relation to size in two patient groups with a respectively low and high probability based on their ages, BMI, and eGFR. Left panel: patients with unilateral AIs, age <63 years, eGFR of ≥80 ml/min/1.73 m^2^, and BMI of ≥28 kg/m^2^ (*n* = 138). The probability for cortisol_DST_ ≥50 nmol/L is below 10% in patients with AIs 10–12 mm in size and approximately 30% in patients with AIs larger than 30 mm. Right panel: patients with unilateral AIs, age ≥63 years, eGFR of 30–80 ml/min/1.73 m^2^, and BMI of <28 kg/m^2^ (*n* = 151). The probability for cortisol_DST_ ≥50 nmol/L is approximately 30% in patients with AIs 10–12 mm in size and approximately 70% in patients with AIs larger than 30 mm. The figures are calculated with restricted cubic splines and are not adjusted for age, BMI, and eGFR.

To visualize the importance of age, BMI, and eGFR in the probability of having cortisol_DST_ ≥50 nmol/L, we created two groups with unilateral AIs and low and high probabilities. Patients <65 years old, eGFR of ≥90, and BMI of >25 were supposed to have a low probability, and patients with age 70–85 years, eGFR of <90 and, BMI of ≤25 were expected to have a high probability. **Figure 9** shows the probability of having cortisol_DST_ ≥50 nmol/L in relation to the size of the AI in the two groups.

### Possible adjustments for differences in age, BMI, and eGFR for the cutoff level for cortisol_DST_

In **Table 5**, we give suggestions for age, BMI, and the eGFR-specific cutoff for elevated levels of cortisol_DST_, which should lead to suspicion of MACS. The characteristics of the patients divided by age-, BMI-, and eGFR-adjusted cutoff levels are presented in **Table 6**. Applying these adjustments in the cutoff level, 34% of the patients were considered to have elevated cortisol_DST_, compared to 46% when 50 nmol/L was used. Thus, approximately 25% of the patients with cortisol_DST_ ≥50 nmol/L were categorised as having normal cortisol_DST_ when the age-, BMI-, and eGFR-specific adjustments were applied. The differences in the size of the AI, the proportion of the bilateral AI, and the ACTH levels between the groups were similar or possibly slightly larger compared to the differences seen using the cutoff level of 50 nmol/L on all patients (see **Table 1**).

**Table 5:** Suggested percentual or absolute adjustments of the established cutoff level of 50 nmol/L for considering cortisol_DST_ to be elevated.

	Relative adjustments in cutoff	Absolute adjustments in cutoff
Age 70–<80 years	+10%	+5 nmol/L
Age 80–<90 years	+20%	+10 nmol/L
BMI 20–<25 kg/m^2^	+20%	+10 nmol/L
BMI 15**–**20 kg/m^2^	+40%	+20 nmol/L
eGFR 70–<80 ml/min/1.73 m^2^	+10%	+5 nmol/L
eGFR 60–<70 ml/min/1.73 m^2^	+20%	+10 nmol/L
eGFR 50–<60 ml/min/1.73 m^2^	+30%	+15 nmol/L
eGFR 40–<50 ml/min/1.73 m^2^	+40%	+20 nmol/L

For example, the suggested cutoff for a patient 75 years old with a BMI of 23 kg/m^2^ and an eGFR of 55 ml/min/1.73 m^2^ is 50 + 5 + 10 + 15 nmol/L = 80 nmol/L.

**Table 6:** Characteristics of patients, according to whether they had cortisol_DST_ above the proposed age-, BMI-, and eGFR-adjusted cutoff levels.

Patient characteristics	Cortisol_DST_ below the specific cutoff level (*n* = 744)	Cortisol_DST_ at the specific cutoff level or higher (*n* = 385)
Age (years)	64.8 (56.7–70.6)	66.3 (60.6–73.4)
Females (*n* (%))	444 (60)	230 (60)
BMI (kg/m^2^)	27.6 (24.5–31.6)	27.0 (24.1–31.0)
eGFR (ml/min/1.73 m^2^)	85 (73–≥90)	84 (69–≥90)
Current smoker (*n* (%))	231 (31)	182 (47)
Comorbidities		
Cardiovascular disease (n (%))	146 (20)	95 (25)
Inhalation steroids (*n* (%))	72 (10)	32 (8)
Hormones		
Cortisol_DST_ (nmol/L)	37 (29–46)	87 (68–125)
ACTH^a^ (pmol/L)	3.7 (2.4–5.4)	2.0 (1.1–3.5)
Imaging		
AI size^b^ (mm)	18 (14–24)	25 (19–31)
Bilateral AI (*n* (%))	80 (11)	100 (26)
AI size second AI in bilateral AI (mm)	15 (12–18)	21 (15–26)

a ACTH analyses with the Roche method were available in 601 patients.

b If bilateral AI, the size of the largest AI is given.

### Calculation of ICC

A second cortisol_DST_ was available in 590 (52%) of the patients, and the time for follow-up was 2.1 (1.7–2.5) years. The variance between subjects was 0.328 (0.296–0.364) and for repeated measures, 0.058 (0.047–0.071). Consequently, the ICC was 0.85.

## Discussion

The study aims to explore the associations between cortisol_DST_ and seven variates of interest with three different statistical methods. We found clinically significant associations not only with the sizes of the AIs but also with age, BMI, and eGFR. The consistency of the results across different analysis methods strengthens the findings. Using ML, we detected a nonlinear association between cortisol_DST_ and BMI. These relations may be of importance for an accurate diagnosis of MACS.

We found cortisol_DST_ increased with age in the whole range of ages studied. The regression coefficient for linear regression and the slope of the curve on the partial dependency plots appear to be similar. The positive association between age and cortisol_DST_ has been described in other patient cohorts earlier, both in subjects without and with adrenal incidentalomas (5, 6, 16, 17). The mechanism is most likely decreased HPA-axis suppressiveness with ageing (17).

A negative association between BMI and cortisol_DST_ was found at BMI levels of <30 kg/m^2^ using ML. This negative association may be strongest at BMI levels below 20 kg/m^2^, and more than 70% of these patients have cortisol_DST_ ≥50 nmol/L. Patients with BMI >30 kg/m^2^ had the lowest cortisol_DST_, which was similar to increasing BMI to at least 40 kg/m^2^. The influence of BMI on cortisol after dexamethasone has earlier been studied in numerous studies, but the majority of these have had a limited number of studied subjects, and most often linear models have been used (18). Recently, Ceccato et al. published a study on a larger cohort of subjects and reported that subjects with a BMI of >30 kg/m^2^ had lower levels of cortisol after dexamethasone (8). They also found a reduced cortisol-to-cortisone ratio and suggested that the lower cortisol was because of an influence on 11β-HSD type 1 and type 2 activities. In patients with adrenal incidentalomas, a negative association between cortisol_DST_ and BMI has also been found by Ueland et al. (7) Interestingly, a study by Schorr et al. has reported a u-shaped association between free urinary cortisol and overnight cortisol and BMI (19). The relation to BMI we found using ML was also found using segmented regression and restricted cubic splines.

We found a relatively linear increase in cortisol_DST_ with decreasing renal function starting at eGFR levels of 80 to 90 ml/min/1.73 m^2^. It is generally accepted that cortisol after dexamethasone is elevated in end-stage renal disease, but we found only one study on the effect of minor reductions in kidney function on cortisol after dexamethasone by Cardoso et al. (9, 20) They found that cortisol_DST_ ≥50 nmol/L was more common in grades 2–4 CKD and absent in all 20 patients with CKD 1. CBG levels were unchanged in CKD 2–4, and they suspected the reason was a central derangement of the HPA axis (9).

Finally, women had a higher prevalence of cortisol_DST_ ≥50 nmol/L, but cortisol_DST_ was not obviously higher in females than men. Prete et al. recently reported that women more often than men have cortisol_DST_ ≥50 nmol/L in patients with adrenal incidentalomas, but other studies have reported no difference (6–8). Finally, we found no clinically significant difference in cortisol_DST_ in patients treated with inhalation steroids. A possible minor influence of this treatment on the HPA axis may be masked by the suppression of dexamethasone.

We studied the relation of cortisol_DST_ to the AIs using the whole cohort and defined the second AI in patients with unilateral AIs as 0 mm. Cortisol_DST_ was, as reported earlier, strongly associated with the size of the AI. Furthermore, it was also associated with the size of the second AI, but at sizes below approximately 15 mm, cortisol_DST_ was similar or only marginally elevated compared to patients with unilateral adenomas. This is consistent with the findings by Vassiliadis et al. of a strong relationship between cortisol after dexamethasone and the size of the AI (21).

The results indicate that in a significant proportion of patients with cortisol_DST_ ≥50 nmol/L the elevation is associated with high age, low BMI, or impaired renal function. The bivariate plots developed with ML indicated that cortisol_DST_ varied with age, BMI, eGFR, and the size of the AI independently. Thus, for patients with combinations of these factors such as low BMI and reduced kidney function the associations to cortisol_DST_ may totally be large. The associations we have found to age, BMI, and renal function have earlier been described in subjects without AI (5, 6, 8–11). Therefore, the associations to cortisol_DST_ may be results of age, BMI, and renal function per se and not caused by differences in cortisol secretion by the AI. In addition, the bivariate plots showed that the associations were present also in patients with small AI as most often have limited or no cortisol secretion. We believe that clinicians should take these associations into consideration when they are to decide whether the patients have MACS. Based on the regression coefficients we propose absolute and relative adjustments in the cutoff level for cortisol_DST_ dependent on age, BMI, and eGFR. Using the proposed adjustments, the patients considered to have elevated cortisol_DST_ had larger AI, more often bilateral AI, and lower ACTH. This shows that the proposed adjusted cutoff levels separate patients into groups with different probabilities to have MACS as the cutoff level of 50 nmol/L.

We have some statistical considerations on the results. Differences in gender, age, BMI, eGFR, and treatment with inhalation steroids could explain approximately 13% of the patient’s variation in cortisol_DST_. The calculated ICC, as may be underestimated, suggests that approximately 15% of the variation of cortisol_DST_ is explained by variation in the measurements. Thus, approximately 72% (100%–15%–13%) of the variation is not explained by associations to gender, age, BMI, eGFR, inhalation steroids, and variation in the measurements. This variation may be a result of the fact that only some of the adenomas secrete cortisol, inter-individual differences in cortisol_DST_, and an effect of factors other than age, eGFR, and BMI. There is always a risk of overfitting when using ML models on small datasets. Our dataset is not that small, but increasing its size could possibly decrease the variance and improve the performance of the models. The *r* ^2^ values for the training set (without cross-validation) and the test set for the models are not dramatically different. The consistency of the results for the partial dependency and the feature importance across the various models also indicates that overfitting is not a problem. Once again, we stress that the ML models were primarily used to uncover nonlinear relationships.

The study has limitations. The study is cross-sectional and gives no proof of causality. The associations found could theoretically be explained by the fact that the cortisol secretion from the AIs was linked to age, BMI, and eGFR. The reports of similar associations in subjects without AIs contradict this, and the association was found also in patients with small adenomas with limited cortisol secretion. The descriptive nature and the absence of predefined aims may lead to the results being data-driven, particularly in terms of the discovered cutoff levels. Furthermore, multicollinearity may lead to unstable estimates of each of the variate’s importance. The relatively large number of studied patients reduces these risks. We only studied a few patients with a BMI above 40, and no conclusions about this group of patients are possible. The dataset is not that big, so the ML models might improve if more patients were considered. The estimate for the association between the size of the second AI and the parameter set to 0 in unilateral AIs may not be accurate since this association may not be linear. The suggested adjustments for the cortisol_DST_ cutoff level were arbitrarily chosen and can probably be improved.

In summary, in patients with an AI cortisol_DST_ is associated positively with age, negatively with BMI at levels below 30 kg/m^2^, and negatively with eGFR. According to bivariate posts, these associations seem independent. This may be the explanation for cortisol_DST_ being elevated to 50 nmol/L or higher in approximately 25% of these patients. We suggest age-, BMI-, and eGFR-specific cutoff levels for considering cortisol_DST_ to raise suspicion of MACS to reduce the risk of incorrectly diagnosing MACS.

## Data availability statement

The raw data supporting the conclusions of this article will be made available by the authors, without undue reservation.

## Ethics statement

The studies involving human participants were reviewed and approved by Etikprövningsmyndigheten. Written informed consent for participation was not required for this study in accordance with the national legislation and the institutional requirements.

## Author contributions

Conception and design: HO and MO. Analysis and interpretation of the data: HO and MO. Drafting of the article: HO and MO. Critical revision of the article for important intellectual content: HO and MO. Statistics: HO (conventional statistics) and MO (ML). Obtaining funding: HO. Collection and assembly of data: HO. All authors contributed to the article and approved the submitted version.
